# Expanding the clinical spectrum of CD3γ deficiency: comprehensive characterization of adult-onset disease and integrated reevaluation of all reported patients

**DOI:** 10.3389/fimmu.2026.1889169

**Published:** 2026-08-13

**Authors:** Raphaela Obeng, Abdulwahab Elsayed, Amos Takyi, Sandra von Hardenberg, Faranaz Atschekzei, Torsten Witte, Georgios Sogkas

**Affiliations:** 1 Department of Rheumatology and Immunology, Hannover Medical School, Hannover, Germany; 2 Hannover Medical School, Cluster of Excellence RESIST (EXC 2155), Hannover, Germany; 3 Department of Human Genetics, Hannover Medical School, Hannover, Germany; 4 Biomedical Research in End-Stage and Obstructive Lung Disease Hannover (BREATH), German Center for Lung Research (DZL), Hannover, Germany

**Keywords:** autoimmunity, CD3, CD3G, CD3γ deficiency, common variable immunodeficiency, Evans syndrome, T cell receptor (TCR)

## Abstract

**Objective:**

CD3γ deficiency is an ultrarare autosomal recessive inborn error of immunity characterized by immune dysregulation and variable immunodeficiency. To date, only 16 predominantly pediatric cases have been reported. Here, we describe two additional adult patients with CD3γ deficiency and provide a comprehensive reevaluation of all previously published cases.

**Methods:**

Clinical, immunological, and genetic investigations were performed in two adult patients presenting with immune dysregulation and hypogammaglobulinemia. Whole-genome sequencing was used to identify pathogenic *CD3G* variants, and protein expression was assessed by Western blot analysis. In addition, a literature review of all previously reported cases was conducted.

**Results:**

Both patients presented with humoral immunodeficiency and Evans syndrome responsive to rituximab therapy. One patient, currently the oldest reported individual with CD3γ deficiency, harbored a novel homozygous frameshift variant in *CD3G* (c.213dupA, p.(Trp72Metfs*6)) resulting in complete loss of CD3γ expression. Reevaluation of all reported cases demonstrated marked phenotypic heterogeneity, ranging from isolated autoimmune manifestations to severe early-onset combined immunodeficiency requiring hematopoietic stem cell transplantation. Notably, patients carrying identical deleterious variants exhibited substantial variability in clinical presentation and outcomes, indicating that no obvious genotype-phenotype correlation could be established based on the currently available data.

**Conclusion:**

CD3γ deficiency exhibits a broad and highly variable clinical spectrum extending into adulthood. Immune dysregulation, particularly autoimmune cytopenias, represents a prominent manifestation. Our findings expand the phenotypic spectrum of CD3γ deficiency and emphasize the importance of considering this disorder also in adult patients with hypogammaglobulinemia and autoimmune disease.

## Introduction

The T cell receptor (TCR)/CD3 complex is a multisubunit structure essential for antigen recognition and T cell activation (1). It consists of a clonotypic TCR αβ heterodimer associated with invariant CD3 chains (γ, δ, and ϵ) as well as the ζ-ζ homodimer, which together ensure correct surface expression and intracellular signaling. Each CD3 chain contains one immunoreceptor tyrosine-based activation motif (ITAM) required for signal transduction, whereas the CD3ζ chain contains three ITAMs, thereby playing a central role in amplifying TCR-mediated signaling.

First reported more than three decades ago (2), CD3γ deficiency is an ultra-rare combined immunodeficiency caused by biallelic, most often homozygous, variants in the *CD3G* gene encoding the CD3γ chain of the TCR (3–5). Loss-of-function mutations typically result in absent CD3γ expression, impairing the assembly and cell surface expression of the TCR/CD3 complex. Consequently, defective TCR signaling affects thymic selection and peripheral T cell activation. In contrast to other CD3 chain deficiencies, namely CD3δ, CD3ϵ and CD3ζ, which are associated with severe combined immunodeficiency due to their essential roles in early T cell development and signal transduction, CD3γ deficiency has been reported to manifest as a milder combined immunodeficiency (5). This difference likely reflects the more accessory role of the CD3γ chain in TCR assembly and signaling, whereas CD3δ and CD3ϵ are critical for thymocyte development and CD3ζ provides the majority of TCR-downstream signaling. Accordingly, partial preservation of T cell development and function is typically observed in CD3γ deficiency, often accompanied by autoimmunity.

Here, we describe two novel cases of CD3γ deficiency, including a patient with a previously unreported homozygous deleterious variant in *CD3G*. Further, we comprehensively review the genetic, phenotypic and immunological spectrum of this monogenic disorder.

## Materials and methods

### Genetic analysis

Genomic DNA extraction and whole-genome sequencing (WGS) were performed as previously described (6). Briefly, genomic DNA was isolated from peripheral blood samples collected in ethylenediaminetetraacetic acid (EDTA) tubes. Library preparation was carried out using the Lotus DNA Library Prep Kit (IDT, Leuven, Belgium), and sequencing was performed on the Illumina NovaSeq 6000 platform, generating 150-bp paired-end reads. Sequencing reads were aligned to the human reference genome (UCSC Genome Browser build GRCh37/hg19), and data were processed using the medical genetics Sequence Analysis Pipeline (megSAP, version 0.2-8-ga9d80c2). Variant filtering was restricted to genes associated with inborn errors of immunity (IEI). Identified variants were classified according to the guidelines of the American College of Medical Genetics and Genomics (7). Rare variants were prioritized based on a minor allele frequency (MAF) ≤1% using population databases, including the Exome Aggregation Consortium (ExAC), the 1000 Genomes Project, and the Genome Aggregation Database (gnomAD). Variant interpretation was supported by publicly available databases, including the Leiden Open Variation Database (LOVD) and ClinVar, as well as in silico prediction tools (REVEL, phyloP, SIFT, PolyPhen-2, FATHMM, and CADD).

The study was conducted in accordance with the principles of the Declaration of Helsinki and approved by the Ethics Committee of Hannover Medical School (approval number: 11223_BO_K_2024). The participants provided written informed consent to participate in this study. Written informed consent was obtained from the participants for the publication of any potentially identifiable images or data included in this article.

### Phenotypic analyses of lymphocytes

Peripheral blood mononuclear cells (PBMC) were obtained from heparinized blood samples of healthy consenting donors and patients by centrifugation over a Ficoll-Hypaque gradient. Phenotypic analyses were performed as multicolour immunofluorescence of PBMC, utilizing directly labeled monoclonal antibodies as described previously (8). Briefly, 1x10^5^ to 2x10^6^ cells/well were incubated with murine monoclonal antibodies against the appropriate antigens at an optimal dilution for 20 min at 4 °C. Nonspecific binding was eliminated by mixing the samples with a 1:5 solution of a commercial human IgG (Octagam, Octapharma). Samples were washed three times in PBS/BSA, and at least 10^4^ cells per appropriate gate were analyzed. The following antibodies (all purchased from Biolegend, if not otherwise stated) were used for this study: CD3 FITC (clone SK7, BD Biosciences), CD3 PE-Cy7, CD3 BUV563 (BD Biosciences), abTCR BU711, abTCR APC, gdTCR BUV395 (BD Biosciences), CD4 APC-Cy7, CD4 PerCP, CD8 PE, CD8a Spark Blue 550, CD16 FITC, CD19 BV510, CD21 PE, CD24 FITC, CD27 BUV661 (BD Biosciences), CD28 PE-Cy5, CD28 APC, CD31 FITC, CD38 PECy7, CD45 APC-Cy7, CD45RO PE-Dazzle 594, CD45RO BV421, CD45 BV785, CD45RA V500, CD56 BV421, HLA-DR APC, PD1 V500, IgD PE, IgM Alexa Fluor 647. Each flow cytometric analysis was controlled with appropriate isotype-matched antibodies. CD19+ cells in the lymphocyte gate were subdivided into the following subsets: naive B cells (IgD+, IgM+, CD27-), IgM+ memory B cells (CD27+, IgD+, IgM+), class-switched B cells (CD27+, IgM-, IgD-), plasmablasts (CD19+/dim, CD27++, CD38++), transitional B cells (IgM++, CD38++, CD24+), CD21 low B cells (CD38 low, CD21 low). CD3+ cells in the lymphocyte gate were subdivided into the following subsets: naive CD4+ T cells (CD4+, CD45RA+), recent thymic emigrants (CD4+, CD45RA+, CD31+), CD4+ memory T cells (CD4+, CD45RO+), Follicular like CD4+ T cells (CD4+, CD45RO+, CXCR5+). All analyses were performed with freshly isolated PBMC using a FACSCanto II flow cytometer with Cell Quest software (Becton Dickinson).

### Proliferation assays

Proliferation assays were performed as described previously (9). Briefly, 2x10^5^ PBMC were cultured in RPMI 1640 medium (Biochrom) supplemented with 10% FCS, 1 mM L-glutamine, 50 U/mL penicillin, 0.5 mM sodium pyruvate, and 50 μg/mL streptomycin) for 48 hours with phytohaemagglutinin, concanavalin A, pokeweed mitogen or immobilized CD3 mAb (iCD3). For immobilization 100 µL anti-CD3 (OKT3, 2.5μg/mL; purified from hybridoma supernatant) was adsorbed onto Nunc Maxi-Sorb 96-well plates (Sigma Aldrich) for 60 min at 37 °C. The plate was washed three times. Proliferation of CD4^+^ T cells was assessed after magnetic enrichment of the target cells from frozen PBMC after thawing using the IMag CD4 T Lymphocyte Enrichment Set (Becton Dickinson) and incubating 5x10^4^ cells/well with media, PHA and iCD3, respectively, in triplicates for 72 hours. Radioactive thymidine was added to each well. 24 hours later cells were harvested and incorporated radioactivity was determined using a beta-counter.

### Western blotting

Proteins were isolated from peripheral blood mononuclear cells (PBMCs) obtained from healthy volunteers and the patient using a DNA/RNA/protein purification kit (Macherey-Nagel) according to the manufacturer’s instructions, as described previously (10). Protein concentration was determined, and equal amounts of protein were loaded onto 12% SDS–polyacrylamide gels (12-well, FastGene) and separated by electrophoresis. Following separation, proteins were transferred onto polyvinylidene difluoride (PVDF) membranes (Thermo Scientific) by wet transfer at a constant current of 100 mA for 90 minutes. Membranes were blocked with 5% non-fat dry milk in phosphate-buffered saline (PBS; Merck) containing 0.1% Tween-20 (PBS-T) for 30–60 minutes at room temperature. Membranes were then incubated overnight at 4 °C under gentle agitation with an anti-CD3γ antibody (Proteintech, 84836-2-RR, 1:1000) and HRP-conjugated goat anti-rabbit IgG (Abcam, ab6721). After washing three times with Tris-buffered saline containing 0.1% Tween-20 (TBST), membranes were incubated with horseradish peroxidase (HRP)-conjugated goat anti-rabbit secondary antibody (Abcam) for 1 hour at room temperature. Following additional TBST washes, protein bands were visualized using enhanced chemiluminescence (ECL) substrate (SuperSignal West Dura Extended Duration Substrate, Thermo Fisher Scientific) with a 5-minute exposure. Images were acquired using an Azure c300 imaging system with ROTI-Lumin substrate (Carl Roth). β-actin (HRP-conjugated rabbit monoclonal antibody; Cell Signaling Technology) served as a loading control. Densitometric analysis was performed using ImageJ software.

### Systematic review of published cases with CD3γ deficiency

A systematic literature review was performed in accordance with the Preferred Reporting Items for Systematic Reviews and Meta-Analyses (PRISMA 2020) guidelines (11). PubMed/MEDLINE was searched for all published cases of CD3γ deficiency until March 2026 using the search terms “(CD3gamma deficiency) OR (CD3g deficiency)”. In addition, the reference lists of all eligible publications were manually screened to identify further relevant reports. Reports lacking sufficient clinical or genetic information and publications written in languages that could not be adequately evaluated were excluded. To avoid duplicate inclusion of patients reported in multiple publications, demographic characteristics (country of origin, sex, age at reporting), genetic findings, pedigree information and author lists were compared. Duplicate cases were included only once. Clinical, genetic, immunological, and outcome data were extracted from all eligible reports for integrated analysis. The study selection process is summarized in the PRISMA flow diagram (**Supplementary Figure 1**).

## Results

### Clinical presentation of two adult patients with CD3γ deficiency

**Figure 1:**
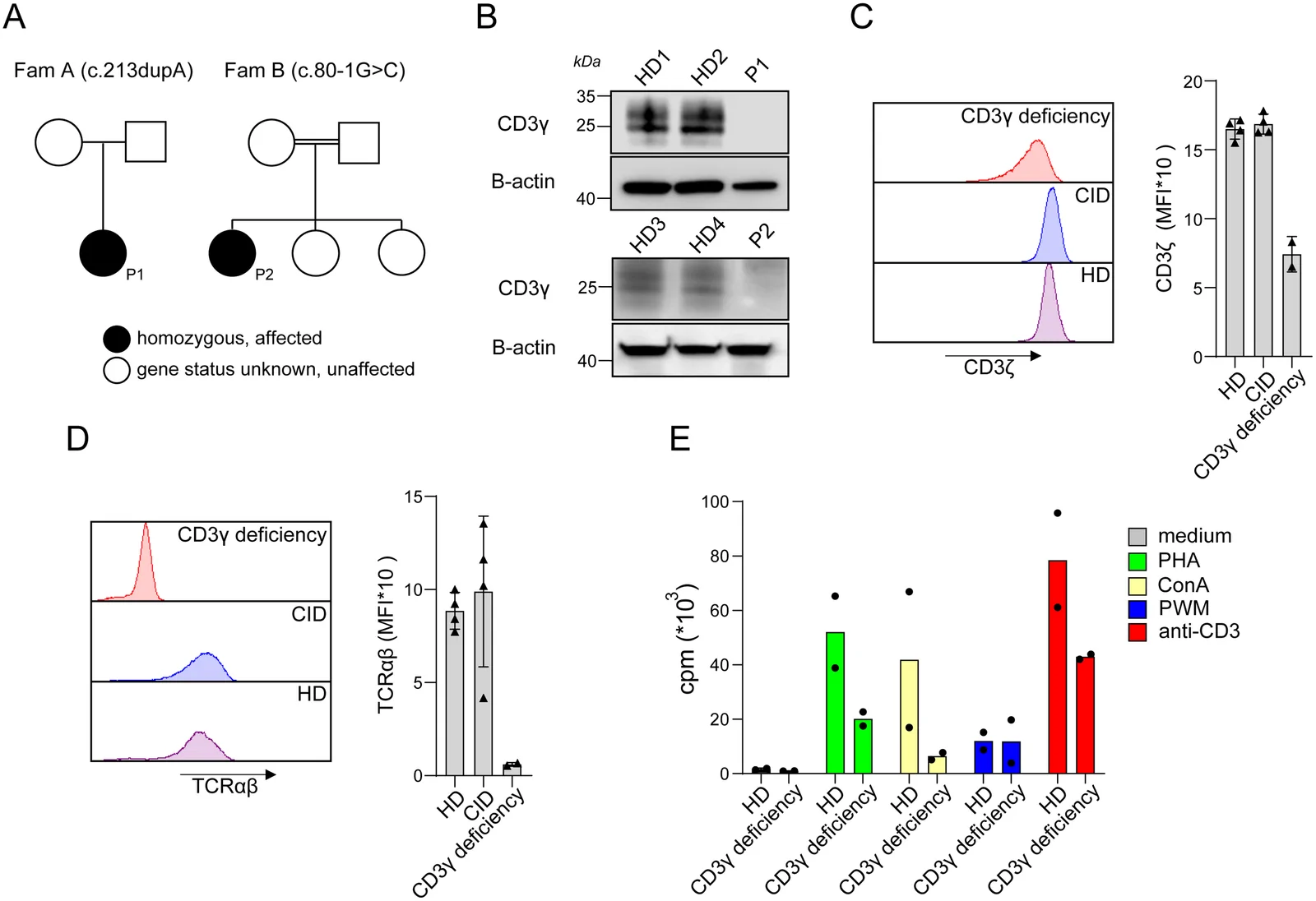
**(A)** Pedigrees highlighting parental consanguinity in family **(B)** P1 and P2 denote the two index patients with CD3γ deficiency. **(B)** Western blot analysis of CD3γ and β-actin expression in peripheral blood mononuclear cells (PBMCs) from the studied patients (P1 and P2) and healthy donors (HD1–HD4). **(C)** Reduced expression levels of CD3ζ and **(D)** T-cell receptor αβ (TCRαβ) in CD3γ deficiency. Representative flow cytometry histograms (left) and summary of median fluorescence intensity (MFI) values (right) from patients with CD3γ deficiency, healthy donors (HD; n = 4), and adult patients with combined immunodeficiency (CID; n = 4) are shown for CD3ζ and TCRαβ expression. **(E)** Reduced proliferative responses to mitogens in CD3γ deficiency. Results are presented as counts per minute (cpm) following stimulation with phytohemagglutinin (PHA), concanavalin A (ConA), pokeweed mitogen (PWM), or immobilized anti-CD3 antibody (anti-CD3).

The first patient (P1) is a 50-year-old woman born to healthy, non-consanguineous Bulgarian parents (**Figure 1A**). Until the age of 37 years, her medical history was unremarkable except for hypothyroidism secondary to Hashimoto thyroiditis. At 37 years of age, she developed generalized lymphadenopathy accompanied by mild pancytopenia. Diagnostic evaluation included three lymph node excisions performed at different time points. Histopathological examination consistently demonstrated reactive lymphoid hyperplasia without evidence of lymphoma or other malignancy. Bone marrow biopsy revealed normal cellularity without signs of hematologic malignancy. Immunological assessment demonstrated markedly reduced serum IgG levels, while IgA and IgM were initially normal (**Table 1**). Immunoglobulin replacement therapy was initiated intravenously and later transitioned to subcutaneous administration, resulting in marked regression of lymphadenopathy. At 42 years of age, the patient developed autoimmune hemolytic anemia (AIHA), initially treated with high-dose prednisolone. However, tapering prednisolone below 40 mg/day resulted in recurrent relapses. During glucocorticoid treatment, she experienced recurrent respiratory infections, including bronchitis and sinusitis, accompanied by progressive IgA and IgM deficiency. Rituximab (RTX; two infusions of 1 g administered two weeks apart) resulted in normalization of hemoglobin levels. One year later, she developed recurrent AIHA with concomitant immune thrombocytopenia (ITP), consistent with Evans syndrome. Reintroduction of RTX as maintenance therapy led to sustained improvement of both hemoglobin and platelet counts. Whole-genome sequencing identified a presumed pathogenic homozygous frameshift variant in *CD3G* (NM_000073.3:c.213dup (p.(Trp72Metfs*6))). Western blot analysis of PBMC-derived protein demonstrated complete absence of CD3γ expression, while flow cytometry revealed reduced expression of the TCR/CD3 complex, confirming the diagnosis of CD3γ deficiency (**Figures 1B–D**).

**Table 1:** Immunological investigations of two adult patients with CD3γ deficiency.

Test:	P1	P2	Normal range
Full blood count:			
WBC (cells/µl)	6,100	5,000	3,900-10,200
Lymphocytes (cells/µl)	2,000	1,350	1,100-4,500
Lymphocytes (% WBC)	40	27	20-44
Monocytes (% WBC)	8.3	7.5	2-9.5
Neutrophils (% WBC)	39.4*	63.5	42-77
Eosinophils (% WBC)	4,6	1,2	0.5-5.5
Basophils (% WBC)	0,9	0,6	0-1.8
CD3^+^ T cells (% lymphocytes)	79	77	55-83
CD4^+^ T cells (% lymphocytes)	46	57	28-57
CD8^+^ T cells (% lymphocytes)	39	16.2	10-39
CD19^+^ B cells (% lymphocytes)	**4**	14	6-19
CD16^+^CD56^+^ NK cells (% lymphocytes)	14	9	7-31
Phenotypic profile of peripheral blood CD3^+^ T cells:			
αβ T cells (% CD3^+^ T cells)	87	92	>85
γδ T cells (% CD3^+^ T cells)	12	7.3	<15
naive CD4^+^ T cells (% of CD4^+^ T cells)	**5**	**8**	49-72
memory CD4^+^ T cells (% of CD4^+^ T cells)	**82.3**	**92**	34-71
RTE T helper cells (% CD4^+^ T cells)	**2.9**	**3**	10-64
PD1^+^ CD4 T cells (% of CD4^+^ T cells)	**69.5**	**19**	2-10.5
HLA-DR^+^ CD4 T cells (% of CD4^+^ T cells)	**27.8**	**18.7**	0.8-4.5
PD1^+^ CD8 T cells (% of CD8^+^ T cells)	**43.8**	**29.2**	0.5-19
HLA-DR^+^ CD8 T cells (% of CD8^+^ T cells)	**35.5**	**19.5**	0.3-6.4
Phenotypic profile of peripheral blood CD19^+^ B cells:			
naive B cells (% B cells)	58	63	29-93
marginal zone B cells (% B cells)	1.1	23	2-25
class-switched B cells (% B cells)	9.3	**1**	3-23
transitional B cells (% B cells)	**5.7**	4	0.6-4.6
plasmablasts (% B cells)	**5.7**	**10**	0.4-3.6
CD21^low^ B cells (% B cells)	1.1	20	1-26
Immunoglobulins:			
IgG (g/l)	**4.6**	**0.49**	7-16
IgG1 (g/l)	**0.08**	**0.55**	4.9-11.4
IgG2 (g/l)	3.83	**0.1**	1.5-6.4
IgG3 (g/l)	**0.12**	**0.07**	0.2-1.1
IgG4 (g/l)	**<0.01**	**<0.01**	0.08-1.4
IgA (g/l)	2.45	**0.07**	0.7-4
IgM (g/l)	1.57	**0.22**	0.4-2.3
IgE (IU/ml)	41.2	25	≤ 100
Complement:			
Total complement activity CH_50_ (%)	54.4	56.2	31.6-57.6
C3c (g/l)	1.36	1.15	0.9-1.8
C4 (g/l)	0.18	0.25	0.1-0.4

HLA, human leukocyte antigen; n.a., not applicable; PD-1, programmed death receptor-1; RTE, recent thymic emigrant; WBC, white blood cells.

*Abnormal values are highlighted in bold.

The second patient (P2) is a 28-year-old woman born to healthy, immunocompetent Moroccan parents who are first-degree cousins (**Figure 1A**). Early development was unremarkable. At the age of 6 years, she developed Evans syndrome, which relapsed at 23 years of age and required treatment with glucocorticoids and high-dose intravenous immunoglobulin (IVIG). Persistent AIHA and ITP refractory to glucocorticoids and IVIG prompted treatment with four weekly infusions of RTX at 26 years of age, resulting in normalization of hemoglobin and platelet counts and allowing discontinuation of glucocorticoid therapy. Infectious complications became apparent in early adulthood. At 21 years of age, the patient developed pneumonia followed by recurrent bronchitis and sinusitis requiring repeated antibiotic therapy. At 22 years of age, she was diagnosed with *Helicobacter pylori*-associated gastric mucosa-associated lymphoid tissue (MALT) lymphoma. Eradication therapy resulted in complete remission. At 27 years of age, she was referred to our outpatient clinic because of severe hypogammaglobulinemia. Initial immunological evaluation was consistent with combined immunodeficiency (CID) (**Table 1**; **Figure 1E**), leading to initiation of immunoglobulin replacement therapy. Whole-genome sequencing identified a homozygous splice-site variant in *CD3G* (NM_000073.3:c.80-1G>C (p.))?, affecting the canonical acceptor splice site of intron 1 and predicted to result in aberrant splicing. This variant has previously been associated with CD3γ deficiency (5). Western blot analysis confirmed complete absence of CD3γ expression (**Figure 1B**).

### Immunological findings in two adult patients with CD3γ deficiency

Initial immunological findings of P1 and P2 are summarized in **Table 1**. Despite normal αβ T-cell counts, both patients demonstrated markedly reduced surface expression of αβTCR and CD3ζ, defining reduced CD3 positivity (**Figures 1C, D**). Reduced expression of the TCR/CD3 complex has similarly been reported in most previously described patients with CD3γ deficiency (2, 4, 5, 12). Reduced proliferative responses to mitogens were observed in both patients, particularly following stimulation with phytohemagglutinin (PHA) and concanavalin A (ConA) (**Figure 1E**). In addition, decreased proportions of naïve CD4^+^ T cells were identified (**Table 1**), further supporting the diagnosis of CID. Similar proliferative defects have been reported in previously published cases (5, 12, 13).

### Literature review and integrative analysis of previously reported CD3γ deficiency cases

**Figure 2:**
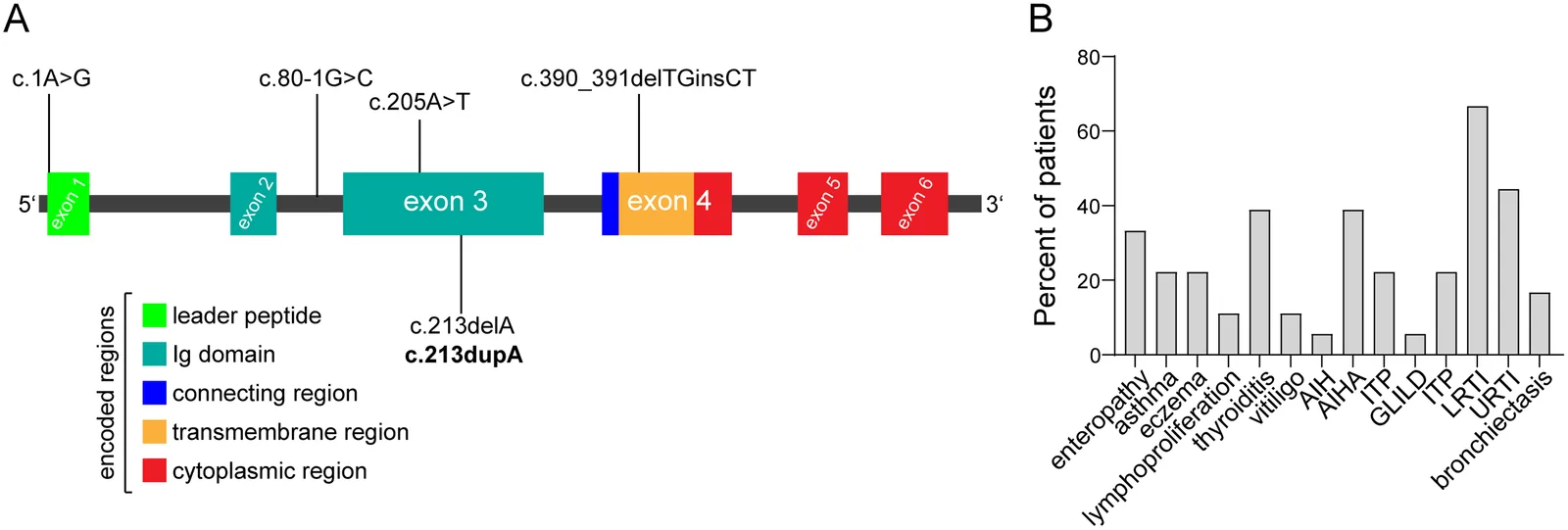
Genetic and clinical spectrum of CD3γ deficiency. **(A)** Summary of all reported pathogenic CD3G variants and their location within the CD3γ protein (adapted from Sonmez et al. (5)). **(B)** Frequency of clinical manifestations among 18 patients with genetically confirmed CD3γ deficiency (percentages are calculated using all 18 reported patients as the denominator).

A review of the literature identified 16 previously reported patients with CD3γ deficiency (**Table 2**) (2–5, 12–17). Including the two patients presented here, affected individuals originated from diverse geographic backgrounds, with parental consanguinity reported in 8 of 18 cases (44.4%). Pathogenic variants included splice-site, nonsense, and frameshift mutations predicted to result in loss of function (**Figure 2A**). Variants were homozygous in all but two reported cases (1/F1–1 and 2/F1-2, **Table 2**). Clinical presentation was highly heterogeneous (**Figure 2B**). Some patients presented in infancy with severe CID characterized by failure to thrive, intractable diarrhea, and severe infections, often resulting in early mortality despite hematopoietic stem cell transplantation (HSCT). In contrast, other individuals exhibited milder phenotypes with survival into adulthood. P1 represents the oldest reported patient with CD3γ deficiency to date. Immune dysregulation emerged as a major clinical feature. The most frequently reported autoimmune manifestations included autoimmune thyroiditis (7/18, 38.9%), AIHA (7/18, 38.9%), and ITP (4/18, 22.2%) (**Figure 2B**).

**Table 2:** Genetic spectrum, clinical and immunological features of 18 patients with CD3γ deficiency.

No.	Family-Pat. Nr./Origin	Sex	Age at last follow-up	*CD3G* variants HGVS nomenclature	Zygo-sity	Main clinical features/Treatment (in case documented)	Immunological features	Vital status/cause of death	Ref.
1	F1-1/Spain	m	31 mo	NM_000073.3:c.80-1G>C (p.)?/NM_000073.3:c.1A>G (p.Met1Val)	Het	SCID-like disease, FTT, intractable diarrhea, AIE, AIHA	low CD3^+^ T-cell percentages; reduced TCR/CD3 expression; low IgG2; multiple autoantibodies, including against intestinal epithelial cells	deceased/viral pneumonia	(2)
2	F1-2/Spain	m	10 y	NM_000073.3:c.80-1G>C (p.)?/NM_000073.3:c.1A>G (p.Met1Val)	Het	chronic respiratory symptoms, diarrhea, asthma, vitiligo, eczema	low CD3^+^/CD8^+^ T cells; reduced TCRαβ/γδ expression; low IgG2; antithyroid autoantibodies	alive	(2)
3	F2-1/Turkey	m	4 y	NM_000073.3:c.205A>T (p.Lys69Ter)	Hom	recur. LRTI, asthma	low T-cell percentages; diminished TCR/CD3 expression; normal IgG/A/M	alive	(12, 14)
4	F3-1/Turkey	m	20 mo	NM_000073.3:c.205A>T (p.Lys69Ter)	Hom	SCID-like disease, diarrhea, pneumonia, otitis, oral candidiasis, diaper dermatitis, perianal fistula/HSCT (twice)	low CD3^+^ T cells; reduced TCRαβ expression; normal IgG/A/M/IgG subclasses; impaired mitogen proliferation	deceased/bilateral pneumonia post HSCT	(12)
5	F3-2/Turkey	m	9 mo	NM_000073.3:c.205A>T (p.Lys69Ter) (presumed)	Hom	SCID-like disease, diarrhea since birth, perianal fistula, oral candidiasis/HSCT	low CD3^+^ T cells; extremely low CD3 expression; normal Ig; impaired mitogen proliferation	deceased/sepsis and diarrhea	(12)
6	F4-1/Turkey	m	10 mo	NM_000073.3:c.205A>T (p.Lys69Ter)	Hom	intractable diarrhea, FTT, IBD, multiple perianal fistulae and fissures, recur. pneumonia episodes, recur. oral candidiasis, skin desquamation/HSCT, IVIG, antifungal and antibacterial prophylaxis	absent CD3^+^ T cells; reduced TCRαβ expression; normal/slightly low Ig; severely impaired proliferation	deceased/respiratory infection post HSCT	(13)
7	F5-1/Turkey	f	12 y	NM_000073.3:c.80-1G>C (p.)?	Hom	Evans syndrome, autoimmune hepatitis, autoimmune thyroiditis, nephrotic syndrome (minimal change disease), hepatosplenomegaly, ALPS/IVIG, steroids, cyclosporin A	low-normal CD3^+^/CD4^+^/CD8^+^; low TCRαβ expression; low IgA/IgG1	alive	(15)
8	F5-2/Turkey	f	20 y	NM_000073.3:c.80-1G>C (p.)?	Hom	Hashimoto thyroiditis, AIHA, short stature	low CD3^+^/CD8^+^; decreased TCR expression; normal IgG/A/M	alive	(15)
9	F6-1/Turkey	f	7 y	NM_000073.3:c.80-1G>C (p.)?	Hom	recur. URTI & LTRI, asthma, bronchiectasis, vitiligo, severe varicella infection, Giardiasis, growth retardation, hyperthyroism, autoimmune thyroiditis	low T cells; reduced TREC; elevated IgG/IgE, normal IgA/IgM; anti-TG, anti-TSHR and anti-TPO pos.; persistent eosinophilia; low PHA-induced proliferation	alive	(16)
10	F6-2/Turkey	m	11	NM_000073.3:c.80-1G>C (p.)?	Hom	atopic dermatitis and pityriasis alba, autoimmune thyroiditis	low T cells; elevated IgE, normal IgG/A/M; anti-TG and anti-TPO pos.; ANA pos.; PHA-induced proliferation 64%	alive	(16)
11	F6-3/Turkey	f	14 mo	NM_000073.3:c.80-1G>C (p.)?	Hom	autoimmune thyroiditis (hypothyroidism)	normal T cells; low TCRαβ/γδ; low IgA, normal IgG/M; PHA-induced proliferation 72%	alive	(16)
12	F7-1/na	na	27 mo	NM_000073.3:c.80-1G>C (p.)?	Hom	*Klebsiella pneumoniae*, MSSA, severe EBV infection, enteropathy, AIHA, GLILD/RTX, sirolimus	normal T cell counts, high B cell and NK cell counts; low IgG and IgM, normal IgA; impaired PHA-/anti-CD3-induced proliferation	alive	(17)
13	F8-1/Taiwan	m	36 y	NM_000073.3:c.213del (p.Lys71fs)	Hom	CVID-like disease, sinopulmonary infections, bronchiectasis, preseptal staphylococcus aureus cellulitis and *E. coli* epididymoorchitis, suspected liver NRH/IVIG, steroids	low CD3/TCRαβ expression; normal Treg, defective B cells; low IgA/M, normal IgG; reduced ConA-induced proliferation, near normal PHA-induced proliferation	alive	(3)
14	F9-1/China	m	10 y	NM_000073.3:c.213del (p.Lys71fs)	Hom	lupus-like disease, thyroiditis (hyperthyroidism), cytopenias, infections/steroids, high dose IVIG	low CD3^+^/CD8^+^/Treg; high IgG/E, slightly increased IgA; ANA (1:320), anti-U1 RNP, anti-SSA, anti-Ro-52 pos.; anti-TG, anti-TSHR and anti-TPO pos.	alive	(4)
15	F10-1/Turkey	m	>30 y	NM_000073.3:c.205A>T (p.Lys69Ter)	Hom	severe varicella infection, respiratory infections, psoriasis, asthma	low CD3^+^ expression, reduced PHA-/ConA-induced proliferation	alive	(5)
16	F11-1/Turkey	m	1 y	NM_000073.3:c.390_391delinsCT (p.Val131Phe)	Hom	recur. gastroenteritis, IBD, sinopulmonary infections, candidiasis/HSCT (twice because of low donor chimerism), steroids (IBD)	normal lymphocytes, very low CD3^+^ expression	alive	(5)
17	F12-1/Bulgaria	f	50 y	NM_000073.3:c.213dup (p. Trp72Metfs*6)	Hom	Hashimoto thyroiditis, lymphadenopathy, AIHA, ITP, alopecia areata/steroids, RTX, SCIG	low B cells; normal T cells and NK cell counts; low IgG, IgA/IgM initially normal; reduced PHA/ConA/anti-CD3-induced proliferation	alive	This report
18	F13-1/Morocco	f	27 y	NM_000073.3:c.80-1G>C (p.)?	Hom	CVID, AIHA, ITP, MALT lymphoma/steroids, high dose IVIG, RTX, SCIG	normal B cell, T cells and NK cell counts, low class-switched memory B cells, reduced PHA/ConA/anti-CD3-induced proliferation; low IgG/A/M	alive	This report

AIHA, autoimmune hemolytic anemia; ALPS, autoimmune lymphoproliferative syndrome; AIE, autoimmune enteropathy; anti-TG, anti-thyroglobulin antibodies; anti-TPO, anti-thyroid peroxidase antibodies; anti-TSHR, anti-thyreotropin receptor antibodies; ConA, concanavalin A; CVID, common variable immunodeficiency; EBV, Epstein-Barr virus; F, family; f, female; FTT, failure to thrive; GLILD, granulomatous-lymphocytic interstitial lung disease; het, heterozygous; hom, homozygous; HSCT, hematopoietic stem cell transplantation; HVGS, Human Genome Variation Society; IBD, inflammatory bowel disease; ITP, immune thrombocytopenia; IVIG, intravenous immunoglobulin; LRTI, lower respiratory tract infections; m, male; MALT, mucosa-associated lymphoid tissue lymphoma; mo, months; MSSA, methicillin-susceptible *Staphylococcus aureus*; na, not available; NRH, nodular regenerative hyperplasia; recur., recurrent; PHA, phytohemagglutinin; Ref., reference; RTX, rituximab; SCID, severe combined immunodeficiency; SCIG, subcutaneous immunoglobulin; TREC, T-cell receptor excision circles; URTI, upper respiratory tract infections; y, years.

With respect to immunological findings, variably reduced T cells were reported in the majority of patients (11/18, 61.1%), while reduced response to mitogens was consistently reported in all 11 tested patients (**Table 2**). The B cell compartment and immunoglobulin profile were heterogeneous, ranging from severe panhypogammaglobulinemia to subclass deficiencies or even normal immunoglobulin levels. Overall, hypogammaglobulinemia or a form of dysgammaglobulinemia was reported in 9/18 patients (50%, **Table 2**). Defects in memory B cells and the presence of multiple autoantibodies were frequently observed.

## Discussion

In this study, we describe two adult patients with genetically confirmed CD3γ deficiency, expanding the known genetic and clinical spectrum of this ultrarare disorder. P1 represents the oldest reported patient with CD3γ deficiency to date, demonstrating that clinically significant disease may remain unrecognized until adulthood. In addition, P2 is, to our knowledge, the first reported patient with *Helicobacter pylori*-associated gastric MALT lymphoma in the context of CD3γ deficiency. Together, these cases further illustrate the remarkable clinical heterogeneity of CD3γ deficiency, extending its spectrum beyond the predominantly pediatric presentations reported to date. However, the causal relationship between CD3γ deficiency and MALT lymphoma remains uncertain, as this observation is based on this single case.

Reevaluation of all reported cases demonstrates a broad spectrum of disease severity ranging from SCID-like presentations to predominant immune dysregulation with adult survival. Notably, even patients harboring identical *CD3G* variants may display markedly different clinical phenotypes, arguing against a straightforward genotype-phenotype correlation. However, given the very limited number of reported patients, the relatively short follow-up and the potential for publication bias, no firm conclusions regarding genotype–phenotype correlations can currently be drawn. Observed phenotypic variability suggests that additional genetic, epigenetic or environmental modifiers likely influence TCR/CD3 complex assembly, signaling capacity and downstream immune regulation.

A consistent immunological finding across many reported patients, including both individuals presented here, is reduced surface expression of the TCR/CD3 complex despite preserved absolute T cell numbers. In contrast, patients with other forms of CID may retain normal αβTCR and CD3ζ expression. Our findings therefore suggest that assessment of αβTCR and CD3ζ surface expression may represent a useful diagnostic marker in patients with suspected CD3γ deficiency. Similarly, impaired proliferative responses to mitogens were observed in both patients and appear to represent a common functional hallmark of the disease. Since mitogens such as PHA and ConA stimulate T cells through crosslinking of TCR-associated glycoproteins, reduced proliferation is likely a direct consequence of impaired TCR/CD3-mediated signaling resulting from defective CD3γ expression (18). An additional observation emerging from the literature review is the possible association between residual TCR/CD3 surface expression and disease severity. Patients with severe early-onset disease frequently exhibited markedly reduced TCR/CD3 expression, raising the hypothesis that residual receptor expression may be associated with clinical severity. However, given the limited number of reported patients, the heterogeneity of flow cytometric methodologies and reporting across studies and the potential for publication bias, this remains a hypothesis-generating observation. Larger, systematically characterized cohorts will be required to determine whether residual TCR/CD3 expression has prognostic value.

A striking feature of CD3γ deficiency is the high prevalence of autoimmunity, especially autoantibody mediated autoimmune manifestations, such as autoimmune cytopenias. Impaired TCR signaling may disrupt thymic selection and permit escape of autoreactive T cells from central tolerance (19). In addition, qualitative defects in regulatory T cell function may contribute to immune dysregulation even when Treg numbers are preserved. Chronic antigenic stimulation secondary to recurrent infections may further amplify these processes and promote autoantibody production.

Our observations also suggest that immunosuppressive treatment may aggravate underlying immunodeficiency in CD3γ-deficient patients. In both P1 and previously reported patient 7/F5-1, worsening immunodeficiency occurred during treatment with high-dose glucocorticoids for autoimmune cytopenias (15). Glucocorticoids are known to suppress TCR signaling at multiple levels, including modulation of downstream signaling kinases and inhibition of TCR-activated transcription factors (20). In the context of an already impaired TCR/CD3 signaling pathway, these effects may further compromise host defense while simultaneously impairing tolerogenic mechanisms. This may partially explain both the severe infectious complications and the limited efficacy of glucocorticoids in controlling autoimmune disease in some patients. Importantly, RTX effectively controlled autoimmune cytopenias in both patients described here and enabled glucocorticoid tapering. Additional immunomodulatory approaches reported in the literature include sirolimus and cyclosporin A. However, these observations are based on a very limited number of patients and should therefore be interpreted with caution.Management of CD3γ deficiency remains challenging and currently ranges from supportive therapy, including immunoglobulin replacement and antimicrobial prophylaxis, to HSCT in severe early-onset disease. Improved understanding of the factors underlying phenotypic variability and disease progression may facilitate risk stratification and individualized therapeutic approaches in the future.

In conclusion, CD3γ deficiency represents a highly heterogeneous inborn error of immunity characterized by variable degrees of immunodeficiency and immune dysregulation. The present study expands the spectrum of adult-onset disease and highlights the importance of considering CD3γ deficiency in patients presenting with autoimmune cytopenias, hypogammaglobulinemia, and impaired TCR/CD3 expression.

## Data Availability

The original contributions presented in the study are included in the article/**Supplementary Material**. Further inquiries can be directed to the corresponding authors.

## Supplementary material

Supplementary Figure 1 Flow diagram illustrating the literature search and study selection process for the systematic review of published cases of CD3γ deficiency based on PRISMA 2000 (PRISMA, Preferred Reporting Items for Systematic Reviews and Meta-Analyses).
